# Education, Sex, and Age Shape Rey Complex Figure Performance in Cognitively Normal Adults: An Interpretable Machine Learning Study

**DOI:** 10.3390/jcm14217562

**Published:** 2025-10-25

**Authors:** Albert J. B. Lee, Benjamin Zhao, James J. Lah, Samantha E. John, David W. Loring, Cassie S. Mitchell

**Affiliations:** 1 Laboratory for Pathology Dynamics, Department of Biomedical Engineering, Georgia Institute of Technology and Emory University, Atlanta, GA 30332, USA; 2 Alzheimer’s Disease Research Center, Department of Neurology, Emory University School of Medicine, Atlanta, GA 30329, USA; jlah@emory.edu (J.J.L.); dloring@emory.edu (D.W.L.); 3 Department of Brain Health, University of Nevada Las Vegas, Las Vegas, NV 89154, USA; samantha.john@unlv.edu; 4 Center for Machine Learning at Georgia Tech, Georgia Institute of Technology, Atlanta, GA 30332, USA

**Keywords:** Rey Complex Figure (CF), Rey–Osterrieth Complex Figure (ROCF), Montreal Cognitive Assessment (MoCA), neuropsychological assessment, demographic differences, machine learning, artificial intelligence

## Abstract

**Background**: Demographic factors such as education, sex, and age can significantly influence cognitive test performance, yet their impact on the Montreal Cognitive Assessment (MoCA) and Rey Complex Figure (CF) test has not been fully characterized in large, cognitively normal samples. Understanding these effects is critical for refining normative standards and improving the clinical interpretation of neuropsychological assessments. **Methods**: Data from 926 cognitively healthy adults (MoCA ≥ 24) were analyzed using supervised machine learning classifiers and complementary statistical models to identify the most predictive MoCA and CF features associated with education, sex, and age, while including race as a covariate. Feature importance analyses were conducted to quantify the relative contributions of accuracy-based and time-based measures after adjusting for demographic confounding. **Results**: Distinct patterns emerged across demographic groups. Higher educational attainment was associated with longer encoding times and improved recall performance, suggesting more deliberate encoding strategies. Sex differences were most apparent in the recall of visuospatial details and language-related subtests, with women showing relative advantages in fine detail reproduction and verbal fluency. Age-related differences were primarily reflected in slower task completion and reduced spatial memory accuracy. **Conclusions**: Leveraging one of the largest reported samples of cognitively healthy adults, this study demonstrates that education, sex, and age systematically influence MoCA and CF performance. These findings highlight the importance of incorporating demographic factors into normative frameworks to enhance diagnostic precision and the interpretability of cognitive assessments.

## 1. Introduction

**Figure 1:**
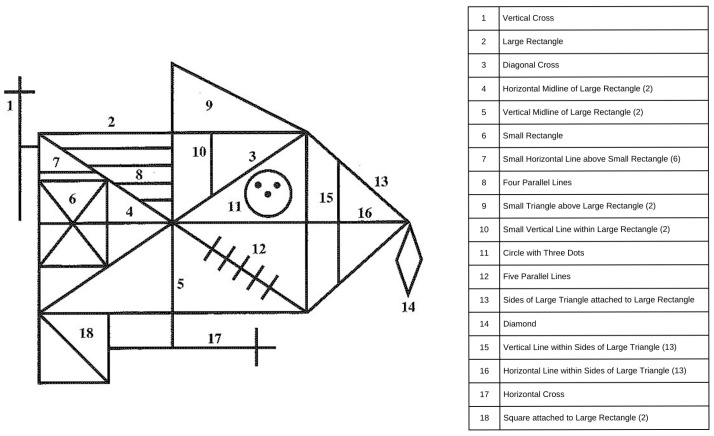
**Rey Complex Figure (CF) scoring items**. The left panel shows the Rey Complex Figure that participants reproduced during the copy, immediate recall, and delayed recall phases of the test. Each numbered element corresponds to a distinct structural component of the figure, summarized in the reference table on the right. The numbered labels are included to help readers identify component features and were not present on the version shown to participants.

**Figure 2:**
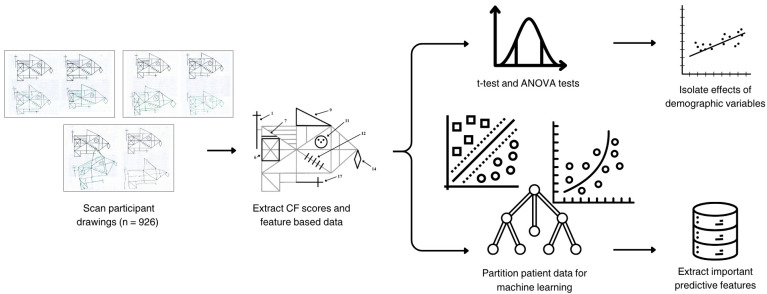
**Pipeline for feature extraction and predictive analysis of CF scores.** The top branch represents the statistical analysis pipeline, which included significance testing via *t*-tests and ANOVA tests using the raw data and subsequent linear regression to isolate the primary demographic variables of interest (education or sex or age). The bottom branch illustrates the machine learning pipeline, which identifies the most important CF or MoCA features to assess the isolated effect of either education, sex, or age.

Developed by André Rey [1], the Rey Complex Figure (CF), also sometimes referred to as the Rey–Osterrieth Complex Figure (ROCF), is a widely used neuropsychological test to assess visuospatial construction, visual memory, and executive functioning. The test involves copying and then recalling a complex geometric design following the copy and after a short delay. The CF is used across both clinical and research settings to assess cognitive functions across a variety of neurological conditions including dementia, traumatic brain injury, and epilepsy. The CF is scored based upon the accuracy and location of 18 individual items. Each unit can have a maximum of two points for a total of 36 points; two points for both an accurate figure and correct location; one point for an accurate figure with incorrect location or inaccurate figure with correct location; 0.5 points for recognizable but inaccurate figure and incorrect location; and 0 points for a missing figure [2]. Figure 1 shows the 18 CF items.

Demographic factors such as age, education, sex, and cultural background are known to significantly influence neuropsychological test performance, and failure to account for these variables can lead to misinterpretation of results and inaccurate classification of cognitive functioning [3,4]. However, few studies have systematically quantified how these demographic variables shape individual CF subscores in cognitively normal adults, limiting the ability to refine normative standards or diagnostic thresholds. Traditional regression approaches often assume linear relationships between demographic factors and test performance, whereas interpretable machine learning (ML) methods can flexibly model nonlinear associations while maintaining transparency in feature-level contributions. Such approaches offer clinical value by identifying which specific cognitive features drive demographic variability—information that can improve interpretation without compromising explainability.

The objective of the present study was to apply interpretable machine learning methods to examine differences in CF performance among cognitively healthy control participants. Previous machine learning CF research has primarily used non-clinically interpretable convolutional neural networks to examine differences between pathological and non-pathological states [5,6]. In this report, we apply interpretable, clinical feature-based machine learning algorithms to assess the impact of demographic features on neuropsychological assessment performance, including sex, education level, and race, in a large sample (n = 926) of cognitively normal participants. By characterizing how demographic factors influence cognitive test components, this study aims to support refinement of diagnostic cutoffs and normative adjustments currently under debate in the cognitive screening literature.

## 2. Materials and Methods

To investigate the influence of demographic variables on cognitive performance, we analyzed data from a large cohort of cognitively healthy adults. Standardized procedures were applied to extract performance metrics from the Rey Complex Figure (CF) and Montreal Cognitive Assessment (MoCA), and both statistical modeling and supervised machine learning approaches were used to identify key predictors. This dual approach ensured that findings were clinically interpretable while leveraging the sensitivity of data-driven methods.

### 2.1. Participant Selection

This was a secondary analysis of previously existing, fully de-identified CF data collected from an existing cohort of participants seen in the clinic at Emory University, Atlanta, GA, USA, as part of the Emory Healthy Brain Study. All participants provided consent according to the Declaration of Helsinki. The original EHBS study protocol was approved by the Internal Review Board of Emory University. The present secondary analysis used only de-identified data and was deemed exempt from additional ethical review.

All CF components were drawn by the participants with pen and paper; no electronic or tablet drawings were utilized. Included participants had no known neurological pathology at the time of CF testing and had Montreal Cognitive Assessment (MoCA) score greater than 24/30. Included demographic participant attributes included sex, age, race, and education. Study exclusion criteria included: (1) incomplete drawings for all three subtests of copy, immediate recall, and delay recall; (2) a MoCA cut-off of <24 utilized based on evidence indicating that the previously recommended threshold of <26 may be overly conservative for classifying normal cognitive functioning [7,8]. The final sample size for this study was 926 cognitively normal participants (n = 926).

### 2.2. CF Scoring

CF reproductions for copy, immediate recall, and delayed recall subtests were independently scored by three experienced research assistants. Immediate recall was obtained approximately 30 s after copy completion, with delayed CF recall obtained approximately 30 min later [7]. One senior clinician who specialized in the grading of the CF was used to consolidate minor differences in scoring and assure score veracity using the traditional Rey scoring system [7]. Although formal inter-rater reliability statistics were not computed, discrepancies were minimal and resolved through consensus to maintain scoring accuracy and standardization across all raters.

There are 18 CF scoring elements that are the same across copy, immediate memory, and delayed memory conditions (Table 1). The features used for grading included the presence or absence of specific features in the CF drawing in the copy subtest (Copy_), the immediate recall subtest (Imm_), or the delayed recall subtest (Del_). Additionally, the drawing times were recorded (in seconds) for each of the 3 subtests. Total CF scores for each CF condition were also used as predictive features.

### 2.3. Feature Inclusion and Data Processing

The primary features included CF copy, CF immediate, and CF delay including completion times for each condition. Additionally, the Montreal Cognitive Assessment and MoCA domain scores were used as predictive features [9]. Only the raw total MoCA score was used as part of the cognitively normal participant selection criterion. Demographic features modeled included sex, age, education years, and race. Sex (male, female) was modeled as a binomial. Age (in years) was binned into 3 groups using age percentile within the population to calculate three relatively equal bin sizes: Age group 1 (45–60 years), Age group 2 (60–67 years), and Age group 3 (67–80 years). Education years were classified based upon highest grade completed (No college, college graduates, post-graduate degree). Race was grouped into Asian, black, or white/Caucasian. Race was not used as an isolated classification target due to substantial class imbalance across groups, which created a high risk of overfitting and misleading performance estimates. However, race was retained as a covariate in regression preprocessing and feature evaluation to account for its potential influence on cognitive performance (see Section 4.5).

### 2.4. Machine Learning to Classify Participant Demographic Features

A combined statistical and machine learning pipeline (Figure 2) was employed to examine associations between Complex Figure (CF) performance and participant demographics (education, sex, and age). Initial significance testing was conducted using *t*-tests and ANOVA, followed by regression to isolate the unique contribution of each demographic attribute and to remove potential confounding. Post-regression *p*-values were checked to confirm that preprocessing effectively controlled for demographic overlap prior to machine learning analysis. To address potential collinearity, we computed pairwise correlations and variance inflation factors (VIFs) across predictors; all VIFs indicated acceptable independence.

Traditional supervised feature-based models—support vector machine (SVM), logistic regression (LR), and random forests—were implemented using scikit-learn with standard default parameters, as these algorithms are well established and require minimal hyperparameter tuning. To ensure generalizability, the data were randomly partitioned into an 80/20 training–testing split, with the test set held out as an independent validation sample. Model fitting, preprocessing, and feature scaling were conducted exclusively on the training data to prevent data leakage. Model performance was assessed using accuracy, precision, recall, and F1-scores to ensure robustness across metrics. Exploratory analyses using k-nearest neighbors (kNN) and principal component analysis (PCA) were conducted to visualize decision boundaries and confirm model separability; as these did not alter the main findings, detailed results are not presented.

To identify the most informative predictors, feature importance values were extracted from Random Forest models using Gini impurity reduction. This approach quantifies how much each feature contributes to decreasing classification error during model training, thereby ranking predictors by their relative contribution. Although alternative approaches such as SHAP or permutation methods were considered, Gini importance was selected for its direct interpretability in clinical contexts and its established use in cognitive feature analysis. Rankings of the top ten features for each demographic classification are reported. All analyses were performed in Python 3.14.0 using the Scikit-learn library.

## 3. Results

The goal of this study was to assess how well demographic features can predict CF performance in normal controls with no overt functional pathology. The end-to-end workflow (Figure 2) first extracted CF subscores and task times, then used classical statistics (*t*-tests/ANOVA and linear models) to quantify raw demographic associations and to regress out non-target demographics before prediction. In parallel, a supervised machine-learning branch trained classifiers on the adjusted feature sets to predict the target demographic and to estimate feature importance.

### 3.1. Cohort Characteristics and Demographic Performance

The cohort’s characteristics are shown in Table 2. Of the eligible controls with intact global cognition (MoCA ≥ 24), the analytic cohort comprised n = 926 participants. Women constituted ~71% of the sample. Most participants self-identified as White, with smaller Black and Asian subgroups. Age was evenly distributed across the three bins (45–60, 60–67, 67–80), and education skewed toward the two higher categories (≥16 years). These distributions demonstrate sufficient representation across the primary demographic variables used for modeling—age, sex, and education. Accordingly, education, sex, and age were retained as the primary independent variables in subsequent analyses. Race was not examined as an isolated variable due to class imbalance but was included as a covariate during regression preprocessing and feature evaluation to account for its potential influence (see Section 4.5).

**Figure 3:**
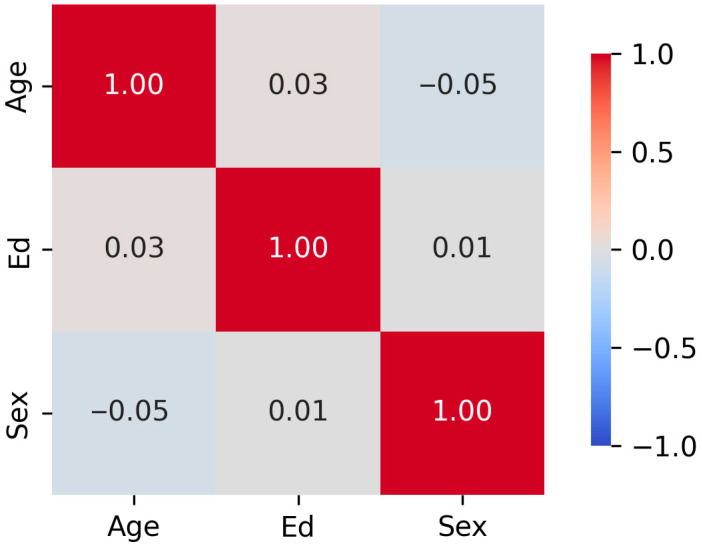
**Correlation Matrix of Demographic Target Relationships**. The correlation matrix shows near-zero associations: ρ (age, education) = 0.03, ρ (age, sex) = −0.05, and ρ (sex, education) = 0.01.

The relationship between demographic targets was evaluated using Variance Inflation Factor (VIF) [10] and Spearman rank correlations. Table 2 shows the calculated VIF and tolerance values, while Figure 3 displays the Spearman rank correlations in a correlation matrix. Given that the VIF values are all less than 5 to 10, and the tolerance values are greater than 0.1 to 0.2 [10], multicollinearity does not exist amongst the demographic variables.

### 3.2. CF Performance by Demographic Group

Demographic performances are shown in Table 3. Across groups, CF Copy scores were narrowly distributed, while Immediate and Delayed recall showed greater spread. Women and men had comparable Copy performance; men showed slightly higher recall sums, whereas women exhibited marginally higher MoCA scores. Age-related trends were evident: earlier age bins showed higher recall sums, with modest variation in task completion times. Increasing education was associated with incrementally higher CF recall and MoCA scores (Table 4).

### 3.3. Statistical Associations of CF Features with Demographics

Statistical analysis (the upper branch of Figure 2) of data was analyzed to examine correlations with demographic attributes. Table 5 summarizes the results of statistical testing, examining the influence of primary demographic variables for which there was a sufficient sample size—education, sex, and age—on CF subscores (Copy_Sum, Imm_Sum, Del_Sum) and MoCA scores, both before and after demographic regression.

Statistical analysis was performed to evaluate associations in the raw data. Several significant associations emerged: education was strongly related to all CF measures and MoCA (all *p* < 0.01), age showed significant effects on immediate and delayed recall and MoCA (*p* < 0.001), and sex was associated with immediate recall, delayed recall, and MoCA (*p* < 0.05).

### 3.4. ML Classification of Demographic Targets

**Figure 4:**
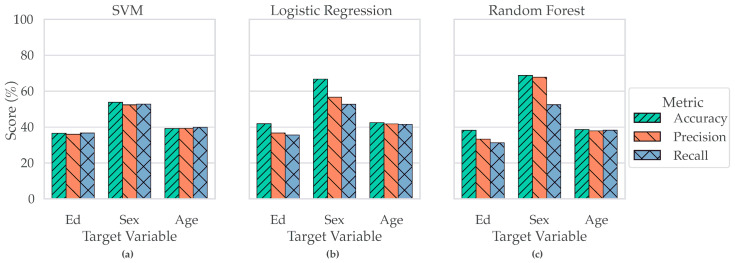
**Classifier Performance by Target and Metric.** Panels (**a**–**c**) show the performance of three classifiers—SVM (**a**), Logistic Regression (**b**), and Random Forest (**c**)—in predicting demographic targets. Each panel displays classifier performance across Accuracy, Precision, and Recall for three target variables: Education (Ed), Sex, and Age. Logistic Regression (**b**) achieved the highest accuracy for predicting both Education and Age, while Random Forest (**c**) outperformed other classifiers in predicting Sex, showing both the highest accuracy and a marked increase in precision.

Machine learning (ML) models were developed to identify patterns associated with individual demographic targets—education, sex, or age—each chosen based on sufficient sample size and class balance. While the models generated demographic predictions, the primary objective was to examine feature importance profiles linked to each target. Race was retained as a covariate to account for potential confounding effects. Classifier performance varied by both target (education, sex, or age) and algorithm (SVM, Logistic Regression, Random Forest) as shown in Figure 4. Logistic Regression achieved the highest accuracy for Education and Age, while Random Forest yielded the best Sex predictions, with the strongest gains in precision. Support Vector Machines performed competitively but did not lead on any target. Together, these results indicate that (i) education- and age-related signal in CF features is captured most effectively by a linear decision boundary after deconfounding, and (ii) sex-related signal benefits from the non-linear partitions afforded by tree-based ensembles.

### 3.5. CF Feature Importance in Predicting Demographic Attributes

Feature-ranking analyses converged on temporal measures as the most consistently informative predictors across tasks. Table 6 shows the top 10 ranked CF features for each classification task: education, sex, and age. The full ranked CF feature importance lists for age, sex, and age are shown in Appendix A Table A1, Table A2 and Table A3, respectively. Copy time and Immediate recall time were the top two features overall, with MoCA close behind. Recall and copy sums contributed secondary information, and MoCA subdomains (e.g., Executive Function) provided additional, target-specific value. The prominence of timing features suggests that processing speed and task efficiency during figure copy and recall carry robust demographic signatures even after statistical deconfounding.

In a cognitively intact control cohort, CF performance exhibits expected demographic gradients at the descriptive level; however, targeted regression effectively removes these associations, enabling fair prediction tasks. Under these conditions, linear models best recover education and age, whereas non-linear ensembles best recover sex. Task-completion times emerge as the most generalizable features, with global cognition (MoCA) and quantitative recall/copy subscores providing complementary signal.

### 3.6. Overlap of Top Predictive Features Across Education, Sex, and Age

**Figure 5:**
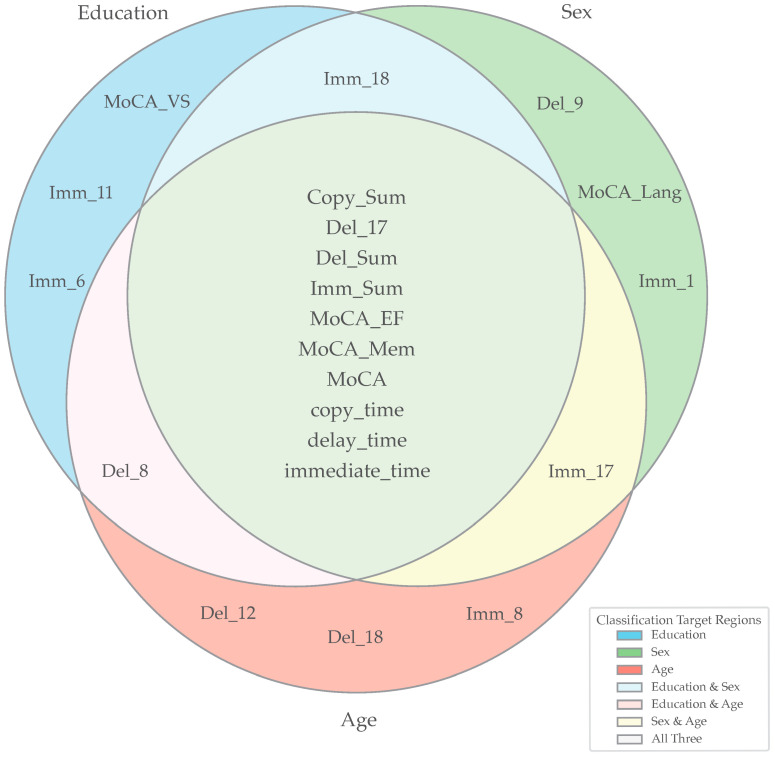
**Overlap of Top 15 Predictive Features Across Classification Targets.** The Venn diagram illustrates the overlap of the top 15 features contributing to the classification of Education, Sex, and Age. Each circle represents the set of features most important for a given demographic classification task, with overlapping regions indicating features shared across tasks. Features placed in the center are predictive across all three targets, while features located in the pairwise overlaps are shared between two demographic targets. The external legend provides color-coded annotations for individual targets (Education, Sex, Age) as well as their intersections (Education and Sex, Education and Age, Sex and Age, and all three combined). Key for abbreviations in this figure: Imm_1 = Imm_Vertical_Cross, Imm_6 = Imm_Small_Rectangle, Imm_8 = Imm_Four_Parallel_Lines, Imm_11 = Imm_Circle_with_Three_Dots, Imm_17 = Imm_Horizontal_Cross, Imm_18 = Imm_Square_attached_to_Large_Rectangle, Del_9 = Del_Small_Triangle_above_Large_Rectangle, Del_12 = Del_Five_Parallel_Lines, Del_18 = Del_Square_attached_to_Large_Rectangle.

Figure 5 presents a Venn diagram illustrating the overlap of the top 15 predictive features across the three demographic classification tasks: education, sex, and age. Several CF and MoCA measures emerged as universally predictive, including Copy_Sum, Imm_Sum, Del_Sum, Del_17, MoCA total, MoCA subdomains (EF, Mem), and task completion times (Copy_time, Imm_time, Del_time). These features clustered in the central intersection, indicating broad predictive utility across all targets. In contrast, a subset of features demonstrated target-specific importance, such as MoCA_VS, Imm_6, and Imm_11 for education; Del_9 and MoCA_Lang for sex; and Del_12, Del_18, and Imm_8 for age. Features like Imm_18 and Imm_17 showed pairwise overlap, linking education with sex and sex with age, respectively. Together, the results highlight both a core set of generalizable predictors and specialized features that drive classification accuracy for demographic groups.

## 4. Discussion

This study investigated how demographic factors influence performance on the Complex Figure (CF) task and related MoCA subtests within a cognitively normal population. By integrating accuracy-based and time-based features, we identified patterns reflecting not only general cognitive ability but also meaningful variations associated with education, sex, and age. Regression analyses further demonstrated that these effects were not statistical artifacts, but rather distinct cognitive profiles across demographic groups. Understanding these differences is clinically relevant, as they provide insight into how demographic factors potentially shape visuospatial processing, encoding strategies, and memory function—ultimately informing both test interpretation and the refinement of normative standards. The following sections examine these demographic influences in detail, beginning with education, followed by sex and age.

### 4.1. Differences Based on Education

When classifying participants by education level, the most predictive features were all time-based or summative metrics: Imm_time, Copy_time, Imm_Sum, MoCA, and Del_time. These variables represent cognitive processing speed, executive planning, and memory consolidation. The time-based metrics, particularly Imm_time and Copy_time, suggest that longer encoding times during figure reproduction are not simply related to age-related slowing, but instead may reflect more deliberate encoding strategies in individuals with higher education, ultimately leading to stronger memory performance. This aligns with evidence showing that greater education enhances executive function and attention to structure in tasks such as CF [11]. On the other hand, summative metrics like Imm_Sum and Del_time emphasize the role of higher education in enhancing both short- and long-term visual memory performance. Previous studies have shown that higher levels of education supports organizational encoding, strategy use, and visual detail tracking, which are all factors that can contribute to stronger CF performance [11,12].

In addition to these predictors (which were found to have high importance when predicting sex and age), three features were uniquely important for predicting education: MoCA_VS, Imm_small_rectangle, and Imm_circle_with_three_dots. MoCA_VS, a measure of visuospatial skill, reflects how educational experience may fine-tune visual construction and mental manipulation skills, consistent with prior studies linking education with visuospatial and design fluency abilities [4]. Notably, previous research has found that individuals with higher levels of education scored higher on MoCA subtests specifically measuring visuospatial abilities and language [13]. This may be because general executive function capacity and working memory tend to be more efficient in individuals with higher levels of education [4,12]. It is important to note that visuospatial subtests are also highly sensitive to early cognitive changes and decline. Kaya et al. [13] found that visuospatial tasks such as clock drawing and trail making were strongly discriminative for mild cognitive impairment (MCI) compared to healthy controls and participants with Alzheimer’s disease (AD). The high importance of Imm_small_rectangle and Imm_circle_with_three_dots is supported by the idea that individuals with higher educational exposure perform better on memory tasks requiring structured encoding of geometric information [11]. These two immediate recall components also measure the early-stage encoding and recall of minor visual components, suggesting that formal education may sharpen the precision and stability of memory traces related to the spatial positioning of smaller shapes within a larger whole.

### 4.2. Differences Based on Sex

Machine learning classification identified CF drawing times as the most prominent difference between male and female participants. This observation is generally consistent with prior work suggesting that copy time may influence recall performance, particularly in delayed recall tests [11]. In contrast to Tremblay et al. [11], however, the current results did not show a clear gender-specific pattern, raising the possibility that time-on-task reflects general cognitive strategies rather than a sex-dependent encoding effect.

Feature importance analyses further indicated that detail-oriented shapes—specifically the vertical cross and the small triangle above the large rectangle—and language ability emerged as distinctive predictors in the sex classification task. These elements require a high degree of precision and visual discrimination, and their predictive importance aligns with prior findings that females often exhibit superior fine motor control and accuracy in visuospatial tasks, particularly those involving visual feedback and structured reproduction [14].

Notably, Del_small_triangle_above_large_rectangle, a delayed recall subscore from the CF, reflects the participant’s ability to retrieve a complex visual element from memory. In this study, Del_small_triangle_above_large_rectangle was more predictive of sex than simpler geometric components such as lines or circles. This pattern supports the hypothesis that high-complexity, centrally located CF features may be especially sensitive to strategic and memory-based sex differences, with females potentially demonstrating advantages linked to attention to fine detail and encoding strategies [11,15].

Similarly, Imm_vertical_cross, which measures immediate recall of one of the cross structures, suggests a sex-related difference in early-stage encoding of detailed visual-spatial relationships. Prior studies indicate that females often approach figure construction more holistically, emphasizing organization and fine detail, which can enhance recall performance for subcomponents such as the vertical cross [16]. This may also reflect differences in encoding strategies, where males tend to perform better on spatial orientation tasks, while females excel in fine motor and precision-based tasks [17].

Lastly, the MoCA_Lang score, reflecting language abilities such as verbal fluency and sentence repetition, was a sex-specific predictor in the classification model. Women frequently outperform men on language-based measures, including verbal memory and fluency [17,18] differences often attributed to both developmental and biological factors such as hemispheric lateralization and neuroendocrine modulation of language networks.

Consistent with prior work, males generally outperform females in gross visuospatial tasks such as mental rotation or global spatial organization, whereas females tend to show relative strengths in segmenting or sequential reproduction tasks [15,16]. These distinctions may partly arise from early social conditioning, with boys more often encouraged to engage in spatially demanding activities such as building or navigation, and girls in tasks emphasizing detailed planning and precision [19,20].

### 4.3. Differences Based on Age

Like the age and education classification tasks, all three drawing times were strongly associated with age. Imm_time, Copy_time, and Del_time reflect processing speed and encoding duration, which are known to decline gradually with age due to slower neural transmission and reduced executive control [11,21]. Older adults may compensate for these changes by allocating more time to encoding; however, this additional time does not always translate into preserved recall performance.

Demographic variables such as education and age have previously been shown to influence MoCA performance, which may help explain why MoCA, MoCA_EF, and MoCA_Mem were among the most predictive features across classification tasks. Standard MoCA cutoff scores can under- or over-estimate impairment depending on demographic characteristics [22]. Even within a cognitively normal sample, these MoCA subscores were closely associated with age classification, consistent with evidence that working memory capacity and episodic recall decline with advancing age [21].

Further feature importance analyses indicated that subscores from both the immediate and delayed recall phases of the CF were strongly age-related. Age has been shown to affect both immediate and delayed recall performance on the CF [11,21], with delayed recall typically showing progressive decline beginning in middle adulthood and becoming more pronounced in the late 50s and 60s [21]. The prominence of Del_five_parallel_lines and Del_square_attached_to_large_rectangle among age-sensitive features supports this pattern: as the brain ages, integrating global structure and relational components—such as aligning parallel lines or reproducing connected shapes—becomes increasingly effortful and error-prone. Meanwhile, Imm_four_parallel_lines as a key predictor highlights that early encoding strategies may begin to deteriorate with age, influencing memory consolidation even over short intervals. This observation aligns with prior research suggesting that older adults often exhibit reduced encoding efficiency, which can affect both working memory and subsequent recall [11]. Age has also been linked to a shift away from structured or hierarchical reproduction strategies, which may contribute to reduced accuracy on spatially complex figure elements, including clusters of lines [15].

### 4.4. Clinical Implications

Although the models in this study were developed for interpretability rather than clinical prediction, the identified demographic effects hold important implications for clinical research and cognitive assessment. Understanding how age, sex, and education influence performance on the CF and related MoCA subtests—and accounting for potential covariates such as race—can improve the interpretation of visuospatial and memory measures commonly used in diagnostic or preclinical screening contexts. Accounting for these demographic influences may also help reduce confounding when using CF performance as a behavioral endpoint in studies of Alzheimer’s disease (AD) or other neurodegenerative conditions.

For instance, demographic-adjusted CF metrics could improve the selection or stratification of participants in clinical trials targeting AD pathology, ensuring that performance differences are not misattributed to disease effects alone. Visuospatial and recall changes measured by the CF are frequently observed within the AD spectrum, but our results suggest that demographic variability must also be considered when interpreting these patterns [23].

Furthermore, integrating CF-derived metrics with other non-invasive biomarkers could enhance interpretability across diverse populations. Blood-based tau phosphorylated at threonine 217 (pTau217), for example, has been shown to distinguish amyloid-positive from amyloid-negative individuals [24], while transcranial magnetic stimulation (TMS) paradigms have detected cholinergic dysfunction associated with AD-related mild cognitive impairment [25]. Coupling such biomarkers with demographically informed CF analyses may strengthen the ability to disentangle disease-related cognitive changes from normative demographic effects, ultimately improving both diagnostic accuracy and trial design.

### 4.5. Limitations and Future Directions

A key limitation of this study is the exclusion of race as a standalone classification target. The sample was predominantly White (91.79%), with limited representation of Black (7.24%) and Asian (<1%) participants. Although class imbalance techniques were applied during preliminary analyses, the small sample sizes for underrepresented groups introduced a high risk of overfitting, inflated performance for the majority class, and misleading conclusions regarding race-based cognitive differences. For this reason, education, sex, and age were selected as the only isolated targets for classification. Race was retained as a covariate during regression preprocessing and feature evaluation to account for its potential influence; however, meaningful classification will require a more demographically balanced dataset. Future studies should prioritize recruitment strategies that enhance racial and cultural representation to enable more inclusive and generalizable analyses.

This study also emphasized clinically interpretable machine learning approaches, using feature-based rather than deep learning models to enhance transparency and clinical relevance [26,27]. While this strategy supports interpretability, it necessarily limits model complexity and potential predictive power. Furthermore, although feature analysis identified CF subscores and MoCA_VS as highly predictive of education, poor performance on these subtests should not be interpreted as impairment in the specific domains they measure, as results may also reflect task familiarity or test-taking strategies [28]. These findings should therefore be viewed as demographic-related performance differences, not diagnostic indicators. Additionally, MoCA subscores do not contribute equally to classification accuracy [29], reinforcing the need for cautious interpretation in clinical and research contexts.

Given the cross-sectional design of this study, it was not possible to determine whether the observed demographic influences on CF and MoCA performance remain stable over time. Future research should adopt longitudinal designs to assess this stability and to evaluate whether demographic characteristics might serve as early indicators of cognitive change or progression toward MCI.

Finally, CF performance was evaluated using traditional pen-and-paper drawings, which likely reduced potential confounding effects related to older participants’ lack of familiarity with digital media. However, as future digital tablet-based assessments become more common in clinical and research settings, future studies should investigate automated scoring methods, deep learning approaches, and pixel-level preprocessing (e.g., pen pressure, line trajectory) to improve measurement precision, sensitivity, and scalability. Future digital CF implementations—with real-time stroke capture and sequencing—would also allow finer-grained evaluation of strategic versus compensatory behavior.

## 5. Conclusions

This study leveraged one of the largest reported samples of cognitively healthy adults (n = 926) and applied clinically interpretable, supervised machine learning methods to examine how education, sex, and age influence performance on the MoCA and CF assessments. Feature importance analyses indicated that higher educational attainment was associated with more deliberate encoding strategies and enhanced memory consolidation, while sex differences were most apparent in the recall of complex visuospatial features and language-related tasks. Age-related effects were characterized primarily by slower processing speed and reduced spatial memory recall accuracy. Collectively, these findings highlight the importance of considering demographic influences when interpreting cognitive test performance, supporting ongoing efforts to refine normative standards and enhance the clinical interpretability of MoCA and CF outcomes across diverse populations.

## Figures and Tables

**Table 1:** Copy, immediate recall, and delayed recall subscores with coded CF component identifiers. The table lists the conversion of Rey Complex Figure (CF) subscores across the three test phases: copy, immediate recall, and delayed recall. Numbers in parentheses correspond to the coded component identifiers shown in Figure 1. In the dataset and results, each CF subtest is denoted as X_, where X is replaced by Copy_, Imm_, or Del_, referring to the respective CF subtests.

Coded Variable X (Copy_, Imm_, Del_)	Actual CF Subscore
X_1	Vertical Cross
X_2	Large Rectangle
X_3	Diagonal Cross
X_4	Horizontal Midline of Large Rectangle (2)
X_5	Vertical Midline of Large Rectangle (2)
X_6	Small Rectangle
X_7	Small Horizontal Line above Small Rectangle (6)
X_8	Four Parallel Lines
X_9	Small Triangle above Large Rectangle (2)
X_10	Small Vertical Line within Large Rectangle (2)
X_11	Circle with Three Dots
X_12	Five Parallel Lines
X_13	Sides of Large Triangle attached to Large Rectangle
X_14	Diamond
X_15	Vertical Line within Sides of Large Triangle (13)
X_16	Horizontal Line within Sides of Large Triangle (13)
X_17	Horizontal Cross
X_18	Square attached to Large Rectangle (2)

**Table 2:** VIF and tolerance values for demographic variables.

Variable	VIF	Tolerance
Age	1.142	0.876
Sex	1.135	0.881
Education	1.110	0.901

**Table 3:** Demographic features and sample sizes.

Demographic Feature	Sample Size (N)	% of Study
Male	271	29.27
Female	655	70.73
White	850	91.79
Black	67	7.24
Asian	9	0.97
Age 1 (45–60 years)	327	35.31
Age 2 (60–67 years)	326	35.21
Age 3 (67–80 years)	273	29.48
Education 1 (10–16 years)	152	16.41
Education 2 (16–18 years)	367	39.63
Education 3 (18–22 years)	407	43.95
Total	926	

**Table 4:** Descriptive statistics of CF scores, subtest scores, and subtest drawing times (copy, immediate recall, and delayed recall) across demographic groups. The table presents the mean standard deviation for Copy Sum, Immediate Sum, Delayed Sum, and MoCA scores, along with average completion times (in seconds) for the copy, immediate recall, and delayed recall tasks. Data are stratified by sex, race, age group, and education level.

Demographic Group	Copy Sum	Immediate Sum	Delayed Sum	MoCA	Copy Time (s)	Immediate Time (s)	Delay Time (s)
Male	32.45 ± 3.08	18.70 ± 6.58	17.67 ± 6.48	26.79 ± 1.69	164.48	129.79	105.58
Female	32.21 ± 3.22	17.91 ± 6.05	16.78 ± 6.30	27.31 ± 1.78	160.00	129.69	103.14
Age 1	32.51 ± 3.12	19.34 ± 6.19	18.10 ± 6.34	27.48 ± 1.77	166.28	135.64	106.66
Age 2	32.23 ± 3.22	18.00 ± 6.06	16.99 ± 6.19	27.01 ± 1.77	156.53	125.61	102.65
Age 3	32.08 ± 3.21	16.89 ± 6.20	15.84 ± 6.39	26.95 ± 1.73	161.06	127.53	101.93
Education 1	31.43 ± 3.42	16.26 ± 6.53	15.47 ± 6.95	26.55 ± 1.73	161.53	128.22	102.30
Education 2	32.42 ± 3.08	18.49 ± 6.15	17.40 ± 6.30	27.16 ± 1.77	159.32	129.29	105.14
Education 3	32.48 ± 3.14	18.54 ± 6.04	17.30 ± 6.11	27.39 ± 1.74	163.02	130.67	103.27

**Table 5:** *p*-values from statistical tests assessing the relationship between demographic variables (education, sex, age) and CF subscores (Copy_Sum, Imm_Sum, Del_Sum, and MoCA) in the raw data. The raw data *p*-values show several statistically significant relationships (*p* < 0.05), particularly between education and all CF scores, as well as between age and immediate/delayed recall and MoCA.

Independent Variable	Dependent Variable	Raw Data *p*-Value
Education	Copy_Sum	0.0027
	Imm_Sum	0.0006
	Del_Sum	0.0097
	MoCA	9.9 × 10^−7^
Sex	Copy_Sum	0.2671
	Imm_Sum	0.0471
	Del_Sum	0.0331
	MoCA	0.0001
Age	Copy_Sum	0.0747
	Imm_Sum	4.85 × 10^−7^
	Del_Sum	6.49 × 10^−6^
	MoCA	0.0001

**Table 6:** The table lists the ten highest-ranked features from random forest classifiers trained to predict education, sex, and age. Feature ranks were derived from model-specific importance scores, where lower rank values indicate greater importance. The “Sum of Ranks” column aggregates feature ranks across all three tasks to highlight features consistently important for prediction. Notably, Copy_time, Imm_time, and MoCA emerged as the three most universally influential features across demographic target classification.

Feature	Rank in Ed	Rank in Sex	Rank in Age	Overall Rank
Copy_time	2	2	2	1
Imm_time	1	5	1	2
MoCA	4	1	4	3
Del_time	5	6	3	4
Imm_Sum	3	8	5	5
Del_Sum	7	4	6	6
MoCA_EF	8	3	8	7
Copy_Sum	6	7	7	8
MoCA_Mem	9	10	9	9
Del_17	15	13	10	10

## Data Availability

Original data requests may be made available upon reasonable request to D.L. and J.J.L. with appropriate approvals.

## Appendix A

**Table A1:** Ranked feature importance for predicting education classification. Effects of age and sex were regressed to enable isolated evaluation of education, but race is kept as a covariate. Features are ranked from most important (Rank 1) to least important (Rank 68) based on their contribution to model performance using a Random Forest classifier.

Feature	Importance Rank
Imm_time_adj	1
Copy_time_adj	2
Imm_Sum_adj	3
MoCA_adj	4
Del_time_adj	5
Copy_Sum_adj	6
Del_Sum_adj	7
MoCA_EF_adj	8
MoCA_Mem_adj	9
Imm_6_adj	10
Imm_18_adj	11
Imm_11_adj	12
MoCA_VS_adj	13
Del_8_adj	14
Del_17_adj	15
Del_12_adj	16
Imm_17_adj	17
Imm_1_adj	18
MoCA_Lang_adj	19
Imm_14_adj	20
Imm_4_adj	21
Imm_8_adj	22
Del_6_adj	23
Del_18_adj	24
Imm_12_adj	25
Del_9_adj	26
Del_14_adj	27
Imm_9_adj	28
Del_11_adj	29
Imm_16_adj	30
Imm_3_adj	31
Del_1_adj	32
Del_4_adj	33
Imm_5_adj	34
copy_7_adj	35
copy_1_adj	36
copy_6_adj	37
copy_9_adj	38
Del_13_adj	39
Imm_7_adj	40
Imm_13_adj	41
Del_16_adj	42
Del_3_adj	43
Del_5_adj	44
copy_3_adj	45
Del_7_adj	46
Imm_15_adj	47
Del_10_adj	48
Del_15_adj	49
Imm_2_adj	50
copy_14_adj	51
copy_18_adj	52
copy_11_adj	53
Hand_adj	54
copy_13_adj	55
copy_2_adj	56
copy_5_adj	57
Del_2_adj	58
Race_adj	59
Imm_10_adj	60
copy_16_adj	61
copy_8_adj	62
copy_12_adj	63
copy_17_adj	64
copy_4_adj	65
copy_15_adj	66
copy_10_adj	67
MoCA_Orient_adj	68

**Table A2:** Ranked feature importance for predicting sex classification. Effects of education and age were regressed to enable isolated evaluation of sex, but race is kept as a covariate. Features are ranked from most important (Rank 1) to least important (Rank 68) based on their contribution to model performance using a Random Forest classifier.

Feature	Importance Rank
MoCA_adj	1
Copy_time_adj	2
MoCA_EF_adj	3
Del_Sum_adj	4
Imm_time_adj	5
Del_time_adj	6
Copy_Sum_adj	7
Imm_Sum_adj	8
MoCA_Lang_adj	9
MoCA_Mem_adj	10
Imm_1_adj	11
Imm_17_adj	12
Del_17_adj	13
Imm_18_adj	14
Del_9_adj	15
Imm_9_adj	16
Del_8_adj	17
Imm_14_adj	18
Imm_8_adj	19
Del_3_adj	20
Del_18_adj	21
Del_1_adj	22
Del_14_adj	23
Imm_6_adj	24
Del_11_adj	25
Del_6_adj	26
Del_12_adj	27
copy_9_adj	28
Imm_13_adj	29
Imm_5_adj	30
Del_2_adj	31
Imm_11_adj	32
Del_16_adj	33
Del_7_adj	34
copy_7_adj	35
Del_13_adj	36
MoCA_VS_adj	37
Imm_15_adj	38
Imm_12_adj	39
Imm_2_adj	40
Imm_3_adj	41
Imm_16_adj	42
copy_1_adj	43
Imm_7_adj	44
copy_6_adj	45
copy_2_adj	46
Del_5_adj	47
Del_4_adj	48
Imm_4_adj	49
copy_3_adj	50
Hand_adj	51
copy_15_adj	52
Imm_10_adj	53
Race_adj	54
copy_18_adj	55
Del_15_adj	56
copy_13_adj	57
copy_5_adj	58
MoCA_Orient_adj	59
Del_10_adj	60
copy_11_adj	61
copy_8_adj	62
copy_14_adj	63
copy_17_adj	64
copy_12_adj	65
copy_16_adj	66
copy_10_adj	67
copy_4_adj	68

**Table A3:** Ranked feature importance for predicting age classification. Effects of education and sex were regressed to enable isolated evaluation of age, but race is kept as a covariate. Features are ranked from most important (Rank 1) to least important (Rank 68) based on their contribution to model performance using a Random Forest classifier.

Feature	Importance Rank
Imm_time_adj	1
Copy_time_adj	2
Del_time_adj	3
MoCA_adj	4
Imm_Sum_adj	5
Del_Sum_adj	6
Copy_Sum_adj	7
MoCA_EF_adj	8
MoCA_Mem_adj	9
Del_17_adj	10
Imm_17_adj	11
Del_18_adj	12
Del_8_adj	13
Del_12_adj	14
Imm_8_adj	15
Imm_6_adj	16
Imm_12_adj	17
Imm_11_adj	18
Del_6_adj	19
Del_9_adj	20
Imm_18_adj	21
MoCA_Lang_adj	22
Imm_14_adj	23
Del_3_adj	24
Del_14_adj	25
Imm_3_adj	26
Imm_1_adj	27
Imm_13_adj	28
Del_7_adj	29
Imm_9_adj	30
Del_11_adj	31
Del_16_adj	32
MoCA_VS_adj	33
Del_1_adj	34
Del_13_adj	35
copy_7_adj	36
copy_9_adj	37
copy_1_adj	38
copy_6_adj	39
Imm_7_adj	40
Del_2_adj	41
Del_5_adj	42
Imm_2_adj	43
Imm_16_adj	44
copy_18_adj	45
copy_3_adj	46
Hand_adj	47
Del_4_adj	48
copy_2_adj	49
Imm_4_adj	50
Imm_5_adj	51
Del_10_adj	52
copy_15_adj	53
Imm_15_adj	54
copy_17_adj	55
copy_11_adj	56
Del_15_adj	57
copy_8_adj	58
Imm_10_adj	59
copy_14_adj	60
Race_adj	61
MoCA_Orient_adj	62
copy_13_adj	63
copy_5_adj	64
copy_16_adj	65
copy_12_adj	66
copy_4_adj	67
copy_10_adj	68
